# Prediction of Non‐Remission in a 5‐Year Follow‐Up of Borderline Personality Disorder in Adolescence

**DOI:** 10.1002/pmh.70041

**Published:** 2025-09-27

**Authors:** Erik Simonsen, Martin Vestergaard, Lise Møller, Sune Bo, Mickey Kongerslev, Lene Halling Hastrup, Ole Jakob Storebø, Stig Poulsen, Emma Beck, Mie Sedoc Jørgensen

**Affiliations:** ^1^ Department of Clinical Medicine, Faculty of Health and Medical Sciences University of Copenhagen Roskilde Denmark; ^2^ Mental Health Services East Region Zealand Denmark; ^3^ Department of Psychology University of Copenhagen Denmark; ^4^ Research Unit, Department of Child and Adolescent Psychiatry Copenhagen University Hospital, Region Psychiatry Zealand Denmark; ^5^ Psychiatric Research Unit Mental Health Services Region Zealand Denmark; ^6^ Mental Health Services West Region Zealand Denmark; ^7^ Department of Psychology University of Southern Denmark Denmark; ^8^ Danish Centre for Health Economics (DaCHE) University of Southern Denmark Odense Denmark; ^9^ Private Practice Roskilde Denmark

**Keywords:** adolescents, borderline personality disorder, female, outcome, prediction, prognosis, psychotherapy

## Abstract

In a 5‐year longitudinal follow‐up study of adolescents (*N* = 97) treated for borderline personality disorder (BPD), we examined the characteristics of the persistent BPD group (*N* = 23), the stability of BPD criteria from baseline to 5 years, and the predictors of remission (*N* = 74). Significantly more participants in the persistent BPD group, compared to the remitted group, had depression, bipolar disorder, schizophrenia, or another personality disorder, received psychotropic medication, and were less likely to be in a relationship or in education. All nine diagnostic criteria of BPD exhibited extremely low stability over time across groups. Prior sexual abuse, conduct disorder, and high levels of general BPD psychopathology at baseline predicted persistent BPD at the 5‐year follow‐up. These findings suggest that while the status from late adolescence to young adulthood on each diagnostic criterion for BPD is highly variable over time, clinicians need to be aware that adolescents with high overall levels of BPD symptoms, a record of sexual abuse, and co‐occurring externalizing behaviors are at increased risk of a poor prognosis.

## 1 Introduction

Several longitudinal studies on the course of adult borderline personality disorder (BPD) have shown varied outcomes over time, with some persons fully recovering, others showing symptom improvement, some staying the same, while others yet experience a worsening of BPD symptoms. Two early landmark longitudinal studies in adults, the McLean Study of Adult Development (MSAD) (Zanarini et al. 2003) and the Collaborative Longitudinal Personality Disorder Study (CLPS) (Gunderson et al. 2006), found early symptomatic remission rates from BPD at around 30% 2 years from the initial diagnosis. A recent meta‐analytic review of 11 studies on the long‐term outcome of 837 adults with BPD, including populations from six different countries in Europe, Canada, and Australia, showed a mean remission rate of 60% within 5 to 15 years from baseline (Álvarez‐Tomás et al. 2019). The review further reported that younger age at baseline was associated with an increased likelihood of being in remission at follow‐up. Moreover, BPD features in adolescents seem more variable and malleable compared to adults (Chanen and Thompson 2018). This provides a rationale for efforts towards the early detection of BPD in adolescent populations to ensure that young people receive appropriate treatment, which may pave the way for a more benign course. Early detection and treatment of BPD are also important because co‐occurrence of other mental health disorders and somatic diseases is very common in adults with BPD (Shah and Zanarini 2018; Hastrup et al. 2024), and these seem to play an important role in the course of the disorder in adults (Shea et al. 2004; Crawford et al. 2008). In one study, individuals remitted from BPD experienced a substantial decline in other mental disorders, whereas those who did not remit from BPD maintained stable rates of comorbid disorders (Shah and Zanarini 2018). Early intervention programs are thought to prevent the cascading effects of co‐occurring mental disorders and psychosocial difficulties (Chanen and Thompson 2018).

BPD is a complex personality disorder (PD), which includes a wide range of behavioral, interpersonal problems, affective, and cognitive phenomena. The borderline construct was originally defined as a level of personality organization, then as a syndrome, and finally as a specific disorder defined by nine distinct criteria that were primarily coined by John G. Gunderson (Gunderson 1994; Gunderson 2009). In adults, all BPD criteria were shown to decline at similar rates in the CLPS study (Gunderson et al. 2011), while findings from the MSAD study showed distinct longitudinal patterns of BPD psychopathology, with affective symptoms being the least variable and most persistent (Zanarini 2003). So far, no studies have specifically examined the stability of the BPD criteria from adolescence to adulthood, although the stability of PDs in general has been shown to be quite low throughout adolescence (Chanen et al. 2004; Chanen et al. 2007).

Clinical improvement in the level of BPD symptoms appears to be important for the level of functioning. While social functioning remained overall impaired at 10‐year follow‐up in the CLPS study, the reduction of BPD criteria predicted subsequent improvements in functioning (Gunderson et al. 2011). The MSAD study reported significantly better psychosocial functioning in ever‐remitted compared to the never‐remitted (persistent) participants across several social and vocational variables (Zanarini et al. 2005). Further, in a Danish register‐based study, we found poor vocational outcomes in first‐admitted adult patients with BPD (Hastrup et al. 2020). Individuals with early onset (< 19 years) BPD showed significantly lower educational attainment and employment rates, as well as higher healthcare costs compared to a matched control group (Hastrup et al. 2022), and by the age of 20 years, the patients with BPD were 22 times more likely to be unemployed and 15 times more likely to be on disability pension than controls. Again, these findings underline the importance of early detection and intervention of BPD.

While most studies on the long‐term outcome of BPD have been carried out in adult samples, recent studies in adolescents with BPD have examined predictors of persistent BPD, developmental trajectories of BPD, and change in BPD symptoms under different treatment conditions (Kaess et al. 2024; Dibaj et al. 2024; Chanen et al. 2022; Chanen et al. 2025). Findings suggest that the natural course of BPD is developmentally influenced by age at therapy commencement. For instance, Kaess et al. (2024) found a clearer decrease in BPD criteria in older adolescents compared to younger adolescents. Specialized interventions like dialectical behavior therapy (DBT) and its adolescent adaptation (DBT‐A) have shown to be effective in reducing core BPD features like self‐harm and suicidal ideation. DBT‐A has demonstrated efficacy in reducing self‐harm, both in short term (3‐year follow‐up) and long term (12.4 years follow‐up) (Diaj et al. 2024). In a study of 139 adolescents and young adults, fewer interpersonal problems, fewer co‐occurring PD diagnoses, and lower BPD severity at baseline predicted better functional outcome at 12 months follow‐up (Chanen et al. 2025). A systematic review identified seven categories of risk for persistent BPD in young people with borderline pathology: adverse childhood experiences, BPD symptom profile, associated mental disorders, personality, and current interpersonal context (Hutsebaut and Aleva 2021). Despite these findings, studies on long‐term predictors of remission from BPD in populations diagnosed in adolescents remain limited and are needed. The present study aimed to examine what characterizes individuals with persistent BPD relative to those who have remitted 5 years from initial BPD diagnosis in adolescence. The original sample of 111 adolescents with BPD has previously been described elsewhere (Beck et al. 2020; Jørgensen et al. 2020). At the last 2‐year follow‐up, 26 (32%) of the 97 who attended follow‐up assessments had remitted from BPD based on self‐report (Simonsen et al. 2021). Here, participants with increased self‐reported depression (internalization) and less exposure to physical abuse had increased odds for remission, while those with comorbid oppositional defiant disorder (externalization) at baseline were less likely to remit. We recently reassessed the participants 5 years after inclusion. Of those 97 participants who attended the 5‐year follow‐up, 23 (24%) still fulfilled the criteria for BPD (Jørgensen et al. 2024). However, it remains unknown what characterizes this subgroup of individuals with persistent BPD relative to those who have remitted from BPD, now as they have reached adulthood. The specific aims of the current study were therefore to examine (1) if those with persistent BPD were worse off than those who remitted from BPD 5 years after initial diagnoses, (2) the stability of the BPD criteria from baseline to 5‐year follow‐up, and (3) predictors of persistent BPD relative to remitted BPD. Based on findings from the existing literature (Hutsebaut and Aleva 2021), we hypothesized that BPD severity at baseline, co‐occurring mental health disorders, and childhood adversity would predict persistent BPD.

## 2 Methods

### 2.1 Sample and Procedures

The current 5‐year follow‐up sample consisted of 96 females and one male adolescent from the original 111 participants, who were reassessed 5 years after inclusion in the study (Beck et al. 2020).

### 2.2 Clinical Assessment at Baseline

The baseline BPD diagnostic criteria were assessed using the semi‐structured Childhood Interview for DSM‐IV Borderline Personality Disorder (CI‐BPD) (Zanarini 2003). Interrater reliability (ICC) was 0.99. CI‐BPD was used to determine fulfillment of the criteria for inclusion and for rating of severity pertinent for subsequent assessment, while the presence of other PDs was assessed using the Structured Clinical Interview for DSM‐IV‐Axis II (SCID‐II) (First et al. 2016). Interrater reliability for this interview was found to be moderate in the MGAB trial (kappa = 0.67; (Beck et al. 2020).

Mental state disorders were assessed with the Mini‐International Neuropsychiatric Interview for Children and Adolescents (MINI‐KID 6.0) (Sheehan et al. 1998). The MINI‐KID is a structured diagnostic interview for children aged 6 to 17 years based on 24 DSM‐IV and ICD‐10 criteria. Interrater reliability on MINI‐KID was excellent (kappa = 0.87) in the M‐GAB trial (Beck et al. 2020).

Borderline features were assessed with the BPFS for children (BPFS‐C), and the corresponding parent version BPFS‐Parent (BPFS‐P) (Sharp and Wright 2015). The BPFS‐C is a 24‐item self‐report questionnaire consisting of four subscales with six items per subscale: affective instability, identity problems, negative relationships, and self‐harm. Items are rated on a 5‐point Likert scale ranging from 1 (not at all true) to 5 (always true). Internal consistency on the BPFS‐C was good to excellent, and excellent for the BPFS‐P at baseline in the M‐GAB trial (Beck et al. 2020). Finally, self‐report information on exposure to lifetime sexual or physical abuse was also collected at baseline.

### 2.3 Clinical and Functional Measures at the 5‐Year Follow‐Up and Reliability of Clinical Ratings

Information on the full assessment battery, information about rater and rating integrity included in the 5‐year follow‐up is reported elsewhere (Jørgensen et al. 2024). Mental disorders at follow‐up were assessed with the WHO diagnostic interview Schedules for Clinical Assessment in Neuropsychiatry (SCAN; WHO 1994). DSM‐5 PD was assessed with the Structured Clinical Interview for DSM‐5 Personality Disorders (SCID‐5‐PD; First et al. 2016). SCAN interview showed almost perfect agreement (kappa = 0.91) and for SCID‐5 substantial agreement (kappa = 0.66), in particular for the BPD criteria on SCID‐5 (kappa = 0.95). Participants who met the clinical cut‐off for BPD on the SCID‐5‐PD, describing five or more symptoms of BPD, were labeled as having persistent BPD, while those who described four or fewer symptoms of BPD were considered to be remitted.

General level of functioning was assessed using the Work and Social Adjustment Scale (WSAS; Mundt et al. 2002). The WSAS is a 5‐item self‐report measure of functional impairment in terms of work, home management, social and private leisure, and close relationships. Items are rated on an 8‐point Likert scale ranging from 0 (no impairment at all) to 8 (very severe impairment). A WSAS score below 10 indicates low functional impairment, 10–19 moderate functional impairment, and 20–40 indicates severe functional impairment.

### 2.4 Statistical Analyses

Statistical analyses were conducted in SPSS 28. A *p*‐value of < 0.05 was considered significant. The primary aim of the study was to examine differences in psychopathology and functional level between young adults in remission from BPD compared to those who meet the diagnostic criteria 5 years later, and to explore predictors of persistent BPD. Participants were classified as remitted from BPD if they no longer met the diagnostic criteria for BPD on the SCID‐5 at the 5‐year follow‐up.

Initially, we used Chi‐square to test if the BPD remission group differed from the BPD group at 5‐year follow‐up in socio‐demographic characteristics, current treatment, co‐occurrence on other PDs, mental disorders, and/or functional recovery. To assure sufficient power, the other PDs were grouped into an “other PD than BPD” variable to test differences in comorbid PD between the BPD group and the BPD remission group.

We tested the stability of the nine BPD symptoms from baseline to 5‐year follow‐up using kappa, while the McNemar change test was used to explore if each of the nine BPD symptoms changed significantly from baseline to 5‐year follow‐up.

Predictors of BPD remission were tested with binary logistic regression models with the diagnoses from the MINI‐KID, BPFS‐C, BPFS‐P, CIBPD, socio‐demographics, lifetime sexual and physical abuse, entered as predictors of outcome, controlling for treatment group and age at baseline.

Continuous variables were determined to have a significantly non‐normal distribution if either of the respective standard errors for the skewness or kurtosis were above or below Z ± 1.96 (two‐sided *p* < 0.05). Non‐normally distributed variables were normalized using the Rankit transformation algorithm. The BPFS‐P and the CIBPD score were non‐normally distributed (*p* < 0.05) and were therefore normalized using the Rankit transformation algorithm. The remaining variables all appeared normally distributed.

### 2.5 Data Management and Ethical Considerations

The study was originally approved by the Regional Ethics Committee of Region Zealand (no: SJ‐371). No additional ethical approval was required for this follow‐up study. The 5‐year follow‐up study is registered at the Danish Data Protection Agency (no: REG‐057‐2020).

## 3 Results

As previously reported (Jørgensen et al. 2024), 74 (76.3%) (mean age 21.4 ± 1.2) of the 97 participants were in remission from BPD, while 23 (23.7%) (mean age 21.9 ± 1.1) met diagnostic criteria for BPD (see Table 1). Among the 23 adults who had persistent BPD, significantly fewer were enrolled in education (*X* ^*2*^(1, *N* = 97) = 3.919; *p* = 0.048) and in a relationship (*X* ^*2*^(1, N = 97) = 4.275; *p* = 0.039), while significantly more were in medical treatment with psychotropics (*X* ^*2*^(1, N = 97) = 7.780; *p* = 0.005), were functionally impaired as defined by the WSAS (*X* ^*2*^(1, *N* = 96) = 13.462; *p* < 0.001), and met the criteria for at least one additional PD (*X* ^*2*^(1, N = 97) = 9.984; *p* = 0.002). The BPD group also more frequently met the criteria for depression (X^2^(1, N = 97) = 11.588, *p* = 0.001), schizophrenia (X^2^(1, N = 97) = 8.607, *p* = 0.003), and bipolar disorder (X^2^(1, N = 97) = 13.423, *p* < 0.001) compared to the 74 adults in BPD remission. No significant differences were observed for employment status, receiving psychotherapy, or having anxiety, OCD, dissociative disorder, or a feeding and eating disorder (*p* > 0.05).

**TABLE 1:** Sociodemographic and clinical variables at 5‐year follow‐up for the whole group (*n* = 97), persistent BPD, versus those remitted from BPD.

Measures at follow‐up	Whole group *N* = 97	BPD persisting *N* = 23 (24%)	BPD remitted *N* = 74 (77%)
Age (mean, SD)	21.5 (±1.2)	21.9 (±1.1)	21.4 (±1.2)
In a relationship	56 (58)	9 (39)*	47 (64)
In employment	51 (53)	8 (35)	43 (58)
In education	47 (49)	7 (30)*	40 (54)
In psychotherapy	46 (47)	15 (65)	31 (42)
On psychotropic medication	43 (44)	16 (70)*	27 (37)
Functional recovery	35 (36)	1 (4)*	34 (46)
Other PD than BPD	30 (31)	16 (70)*	24 (32)
Depression	31 (32)	14 (61)*	17 (23)
Schizophrenia	15 (16)	8 (35)*	7 (10)
Bipolar disorder	4 (4)	4 (17)*	0 (0)
Anxiety disorders	36 (37)	11 (48)	25 (34)
OCD	11 (11)	5 (22)	6 (8)
Feeding or eating disorders	13 (13)	4 (17)	9 (12)
Dissociative disorders	3 (3)	0 (0)	3 (4)

* Significant group differences at *p* < 0.05 tested with Chi‐square. BPD versus BPD remitted.

### 3.1 Stability of BPD Criteria

Table 2 displays the frequency distributions for each BPD criterion at the clinical cut‐off at baseline and follow‐up. The prevalence of all nine BPD criteria decreased significantly from baseline to follow‐up (*p* < 0.0001). Kappa values were exceedingly low for all nine BPD criteria, suggesting low stability over time.

**TABLE 2:** Course of borderline personality disorder from baseline to 5‐year follow‐up.

	Baseline^a^	Follow‐up^b^	Absent follow‐up	New cases follow‐up	Repeated cases	Kappa
BPD criteria	*N* (%)	*N* (%)	*N* (%)	*N* (%)	*N* (%)	
Abandonment	64 (66)	33 (34)	40 (41)	9 (9)	24 (25)	0.083
Unstable relationships	85 (88)	25 (26)	62 (64)	2 (2)	23 (24)	0.033
Identity disturbance	73 (75)	14 (14)	61 (63)	2 (2)	12 (12)	0.044
Impulsivity	86 (89)	22 (23)	64 (66)	0 (0)	22 (23)	0.072
Suicidality	90 (93)	23 (24)	67 (69)	0 (0)	23 (24)	0.047
Affective instability	96 (99)	45 (46)	51 (53)	0 (0)	45 (46)	0.018
Emptiness	72 (74)	36 (37)	43 (44)	7 (7)	29 (30)	0.084
Inappropriate anger	80 (83)	18 (19)	63 (65)	1 (1)	17 (18)	0.063
Stress‐related paranoid ideation or dissociation	71 (73)	28 (29)	46 (47)	3 (3)	25 (26)	0.155

*Note:* Because the participants at baseline were below the age of 18 years, none received a diagnosis of antisocial personality disorder.

^a^ Measured with the Childhood Interview for DSM‐IV Borderline Personality Disorder (Zanarini 2003).

^b^ Measured with the SCID‐5‐PD.

### 3.2 Prediction of Remission and Non‐Remission of BPD

Descriptive statistics for the baseline measures for the remitted compared to the non‐remitted group are presented in Table 2. The results of the binary logistic regression analyses of predictors of remission are presented in Table 3. Fulfilling the diagnostic criteria of BPD relative to being in BPD remission 5 years later was predicted by the following variables at baseline: (a) fulfilling the criteria for conduct disorder, (b) self‐reported exposure to sexual abuse, and (c) increased severity of BPD symptoms assessed with the semi‐structured interview CI‐BPD (see Table 4).

**TABLE 3:** Descriptive statistic for baseline measures (*n* = 97).

Measures at baseline	Whole group *N* = 97	BPD persisting N = 23 (24%)	BPD remitted *N* = 74 (76%)
Age (mean, SD)	15.9 (±1.1)	16.2 (±0.9)	15.8 (±1.1)
On psychotropic medication	43 (44)	13 (57)	41 (55)
Attending school	86 (89)	19 (83)	67 (91)
Sexual abuse	15 (15)	7 (30)	8 (11)
Physical abuse	23 (24)	3 (13)	20 (27)
Parent drug/alcohol abuse	35 (36)	10 (43)	25 (34)
Depression	52 (54)	12 (52)	40 (54)
Any anxiety disorder	77 (79)	19 (83)	58 (78)
Panic disorder	39 (40)	10 (43)	29 (80)
Agoraphobia	62 (64)	16 (70)	46 (62)
Social anxiety	48 (49)	13 (57)	35 (47)
Generalized anxiety	23 (24)	5 (22)	18 (24)
OCD	28 (29)	6 (23)	11 (15)
PTSD	11 (11)	3 (13)	8 (11)
ADHD	18 (19)	5 (22)	13 (18)
Conduct disorder	18 (19)	8 (35)	10 (14)
Oppositional disorder	40 (41)	9 (39)	31 (42)
CIBPD (median; Q1–Q3)	16	17 (15–18)	16 (13–17)
BPFS‐P (median; Q1–Q3)	81	78 (66–90)	82 (67–89)
BPFS‐C (mean ± SD)	79.9	80.8 (±10.7)	79.7 (±12.7)

**TABLE 4:** Predictors of BPD compared to BPD remission at baseline.

Measures at baseline	Β	S.E.	Wald	*p*
On psychotropic medication	0.095	0.496	0.037	0.85
Attending school	0.535	0.702	0.580	0.45
Sexual abuse	−1.215	0.601	4.083	**0.043** *
Physical abuse	0.778	0.686	1.285	0.26
Parent drug/alcohol abuse	−0.374	0.499	0.562	0.45
Depression	0.117	0.489	0.057	0.81
Any anxiety disorder	−0.322	0.644	0.250	0.62
Panic disorder	−0.102	0.496	0.042	0.84
Agoraphobia	−0.273	0.530	0.265	0.61
Social anxiety	−0.298	0.493	0.365	0.55
Generalized anxiety	0.131	0.585	0.050	0.82
OCD	0.027	0.561	0.002	0.96
PTSD	−0.427	0.754	0.321	0.57
ADHD	−0.281	0.601	0.218	0.64
Conduct disorder	−1.439	0.591	5.937	**0.015** *
Oppositional disorder	0.000	0.517	0.000	0.99
CIBPD	0.799	0.316	6.376	**0.012** *
BPFS‐P	−0.028	0.240	0.013	0.91
BPFS‐C	0.008	0.020	0.141	0.71

*Note:* The binary logistic regression models were controlled for treatment group and age at baseline.

* Significant group differences at *p* < 0.05.

## 4 Discussion

In this sample of young adults, of which the vast majority met the diagnostic criteria for a diagnosis of BPD in adolescence, 76.3% were in remission from BPD after 5 years. The meta‐analysis by Álvarez‐Tomás et al. (2019) on the long‐term clinical and functional course of BPD in adults indicated a 60% remission rate of BPD. Our findings specifically in this group of adolescents are comparable to the results from a study by Biskin et al. (2011), who found a remission rate of 65% at a 4‐year follow‐up in females diagnosed with BPD before the age of 18 years, and Chanen et al. (2004) similarly found a remission rate of 60% at a 2‐year follow‐up. More recent follow‐up studies of adolescents with BPD pathology within intervention trials have tended to focus on specific psychopathological outcomes, such as self‐harm (Dibaj et al. 2024) or psychosocial and interpersonal functioning (Chanen et al. 2025), rather than remission of the disorder itself as defined by the diagnostic criteria. The sample from the DBT‐A study of self‐harm and suicidal ideation only had 20.5% with BPD, and they were assessed by self‐report. Our study is therefore a novel contribution, since in our opinion, no other study of adolescents with BPD has specifically reported data of remission based on reassessment of diagnostic criteria using semi‐structured diagnostic interviews.

We found that a comorbid conduct disorder, self‐reported exposure to sexual abuse, and high levels of overall BPD psychopathology at baseline predicted having persistent BPD at follow‐up in adulthood. In our previous 2‐year follow‐up study, we found that participants with comorbid oppositional defiant disorder at baseline were less likely to have clinically improved and remitted from BPD (Simonsen et al. 2021). In the current study, having a conduct disorder at baseline predicted non‐remission of BPD at the 5‐year follow‐up. This suggests that while externalizing behaviors, such as oppositional defiant disorder, which are characterized by anger, irritability, arguing, and refusing to comply with rules, may influence the course of BPD in the short run, more severe forms of aggression and anti‐social behavior, as seen in conduct disorder, are more predictive of the long‐term outcome of BPD. Thus, like our findings from the two‐year follow‐up study (Simonsen et al. 2021), the present results indicate that symptoms of externalization are more predictive of poor prognosis relative to internalizing symptoms. As mentioned in the introduction, abnormal personality was one of the six predictors found in the review of risk profiles for BPD prognosis (Hutsebaut and Aleva 2021). Low agreeableness, high neuroticism, and impairment in personality functioning, as rated by the Alternative Model for Personality Disorders (AMPD) Criterion A, were associated with poorer BPD outcomes.

Self‐reported childhood sexual abuse was another significant negative predictor of BPD remission. At the 2‐year follow‐up, we found that physical abuse predicted non‐remission. The harmful long‐term effects of childhood sexual abuse were first reported by Browne and Finkelhor (1986), who found that sexual abuse may lead to fear, anxiety, depression, anger, and hostility, and a range of physical, emotional, sexual, self‐esteem, and social functioning. Judith Herman et al. (1989) reported that both physical and sexual abuse were strongly associated with a diagnosis of BPD, and Johnson et al. (1999) found that sexual abuse was associated with elevated symptom levels of BPD. Stone (1990, 2017) was the first to show the strong negative impact of sexual abuse on therapy outcomes, especially in severe cases of abuse. Zanarini (2000) pointed out that childhood adversity and maltreatment were serious risk factors for the development of BPD, although not independent of genetic factors. Sexual abuse, in particular, may lead to distortions in interpersonal relationships and a pervasive mistrust in other people. In the MSAD study, the presence of sexual abuse was one of the predictors of negative outcomes at the 10‐year follow‐up (Zanarini et al. 2006). Similarly, Biskin et al. (2011) found that persistent BPD was predicted by current depressive episode, lifetime substance abuse, and self‐reported childhood sexual abuse among adolescent girls with BPD. Paris and colleagues (1993) also showed that childhood sexual abuse was more frequent in patients who remained symptomatic of BPD (Paris et al. 1993), a finding later supported by a study of Soloff et al. (2002), reporting that sexual abuse was associated with chronicity and higher levels of suicidality. Our finding that sexual abuse was a strong predictor of a negative outcome specifically underscores the need for trauma‐informed care in traumatized individuals with BPD.

We also found that higher overall severity of BPD symptoms at baseline, assessed with the semi‐structured interview CI‐BPD, predicted persistent BPD after 5 years. In contrast, neither self‐reported nor parent‐reported levels of BPD symptoms at baseline on the BPFS predicted the outcome of BPD 5 years later. This is consistent with our previous lack of findings at the two‐year follow‐up, where self‐reported levels of BPD symptoms at baseline showed no apparent relationship with self‐reported remission from BPD after 2 years, defined by a clinical cut‐off on the BPFS (Simonsen et al. 2021). This indicates that clinical assessment of BPD using a semi‐structured interview has more predictive power than both self‐reported and parent‐reported questionnaires on BPD over the course of 5 years, and therefore, that the semi‐structured interview is a more reliable and valid method of choice. The finding that higher severity of BPD psychopathology in adolescents at baseline is an important predictor of outcome corresponds with results from diagnostic interviews in clinical trials in adults (Shea et al. 2004; Gunderson et al. 2014) and in adolescents (Chanen et al. 2025). Gunderson et al. (2016) also found that more severe BPD psychopathology at baseline was associated with more functional impairment and less quality of relationship for the non‐remitted patients. Our finding of severity as an important predictor supports the focus in ICD‐11 on the rating of severity as the first important step after the assessment of general criteria for PDs (WHO 2024).

We found an overall decrease in all BPD symptoms from baseline to 5‐year follow‐up. This is in accordance with the findings in adult samples, both for the course of PDs in general and in studies specifically on BPD and subsyndromal phenomenology (Grilo et al. 2004; Gunderson et al. 2011; Zanarini et al. 2012). The low kappa coefficient is first explained by the use of different diagnostic instruments but may also express wax and wane and reoccurrence of borderline symptoms over time. The low diagnostic stability of PDs and of PD criteria in general, including for BPD, is expected, as shown in a recent systematic review and meta‐analysis of the stability across different age groups at shorter and longer follow‐up (d'Huart et al. 2023).

Diagnostic stability was originally highlighted as one of the most important validators of a diagnostic construct (Robins and Guze 1970). Recent research has indicated that BPD may not be regarded as a disorder in traditional terms, but rather a disposition to personality pathology in general (the g‐factor) representative of all PDs (Sharp and Wright 2015) and/or even as a specifier for severity as introduced in ICD‐11 (WHO 2024).

This study describes some very interesting findings on predictors of persistent BPD, offering valuable insights to inform clinicians. However, the data is limited by the lack of control over the treatment received. The need for identifying predictors of treatment effects in randomized clinical trials (RCTs) is increasingly recognized as crucial for advancing personalized medicine. Raw data from such trials can serve as a valuable resource for individual patient data (IPD) analyses, offering insights that go beyond the aggregate‐level findings typically reported in published studies. Specifically, examining moderating effects of co‐occurring disorders on BPD severity, such as affective disorders, anxiety disorders, substance abuse, PTSD, eating disorders, and other PDs, is vital for refining treatment strategies and improving patient outcomes. These moderating factors can influence both the severity of BPD and the effectiveness of interventions, underscoring the need for detailed subgroup analyses in IPD research. Understanding these interactions could lead to more tailored treatment approaches and a better understanding of which interventions work best for specific patient profiles (Storebø et al. 2021).

## 5 Conclusion

BPD detected in adolescence shows a high rate of remission of diagnostic criteria after 5 years, with over three‐quarters of participants remitting from BPD. Our findings support the importance of the assessment of severity of BPD in the classification of PDs, as outlined in the forthcoming ICD‐11. For the management of BPD in adolescents, there seems to be a particular need for specialized programs tailored to those with comorbid externalizing disorders and early trauma. Addressing these factors may help mitigate their burden and prevent poor long‐term outcomes.

## 6 Strengths and Limitations

The major strength of the study is its very low attrition rate of 13% at 5‐year follow‐up, and the comprehensive mental disorder evaluation using established semi‐structured interviews accompanied by self‐report instruments. One limitation is the use of different diagnostic instruments over time. We adapted our assessment methods for PDs to reflect the developmental changes that occur with age, since the diagnostic criteria were originally developed for adults with BPD. During the 5 years, participants engaged in different uncontrolled treatments without reliable information, which limited our ability to control for time–treatment interactions and other confounding variables. We used binary logistic regression models to ease comparison with previous studies. Consequently, more advanced statistical techniques might have uncovered multivariate patterns not detected by our basic analytical approach. Moreover, while we critically selected which predictor variables to include in the present study based on prior research, the relatively high number of binary logistic regression analyses increases the risk of false positives. However, we refrained from doing multiple comparison correction due to the exploratory design of our study.

## Conflicts of Interest

The authors declare no conflicts of interest.

## Data Availability

The data that supports the findings of this study are available in the supplementary material of this article.

## References

[pmh70041-bib-0001] Álvarez‐Tomás, I. , J. Ruiz , G. Guilera , and A. Bados . 2019. “Long‐Term Clinical and Functional Course of Borderline Personality Disorder: A Meta‐Analysis of Prospective Studies.” European Psychiatry 56, no. 2019: 75–83. 10.1016/j.eurpsy.2018.10.010. 30599336

[pmh70041-bib-0002] Beck, E. , S. Bo , M. S. Jørgensen , et al. 2020. “Mentalization‐Based Treatment in Groups for Adolescents With Borderline Personality Disorder: A Randomized Controlled Trial.” Journal of Child Psychology and Psychiatry, and Allied Disciplines 61, no. 5: 594–604. 10.1111/jcpp.13152. 31702058

[pmh70041-bib-0003] Biskin, R. S. , J. Paris , J. Renaud , A. Raz , and P. Zelkowitz . 2011. “Outcomes in Women Diagnosed With Borderline Personality Disorder in Adolescence.” Journal of the Canadian Academy of Child and Adolescent Psychiatry 20, no. 3: 168–174. 21804844 PMC3143691

[pmh70041-bib-0004] Browne, A. , and D. Finkelhor . 1986. “Initial and Long‐Term Effects Sexual Abuse. A Review of the Research.” In A Sourcebokk on Child, edited by D. Finklel , 143–179. Sage Publications.

[pmh70041-bib-0005] Chanen, A. M. , H. Andrewes , K. Nicol , et al. 2025. “Baseline Correlated of Functional Impairment at 12 Month in Young People with Borderline Personality Disorder: Findings From the MOBY Randomised Controlled Trial.” European Child and Adloscent Psychiatry 2025. 10.1007/s00787-025-02742-5. PMC12647327 40434712

[pmh70041-bib-0006] Chanen, A. M. , J. K. Betts , H. Jackson , et al. 2022. “Effect of 3 Forms of Early Intervention for Young People with Borderline Personality Disorder: The MOBY Randomized Clinical Trial.” JAMA Psychiatry 79, no. 2: 109–119. 34910093 10.1001/jamapsychiatry.2021.3637 PMC8674805

[pmh70041-bib-0007] Chanen, A. M. , H. J. Jackson , P. D. McGorry , K. A. Allot , V. Clarkson , and H. P. Yuen . 2004. “Two‐Year Stability of Personality Disorder in Older Adolescent Outpatients.” Journal of Personality Disorders 18, no. 6: 526–541. 15615665 10.1521/pedi.18.6.526.54798

[pmh70041-bib-0008] Chanen, A. M. , L. K. McCutcheon , M. Jovev , H. J. Jackson , and P. D. McGorry . 2007. “Prevention and Early Intervention for Borderline Personality Disorder.” Medical Journal of Australia 187, no. S7: S18–S21. http://www.ncbi.nlm.nih.gov/pubmed/17908019. 17908019 10.5694/j.1326-5377.2007.tb01330.x

[pmh70041-bib-0009] Chanen, A. M. , and K. N. Thompson . 2018. “Early Intervention for Personality Disorder.” Current Opinion in Psychology 21: 132–135. 10.1016/j.copsyc.2018.02.012. 29533848

[pmh70041-bib-0010] Crawford, T. N. , P. Cohen , M. B. First , A. E. Skodol , J. G. Johnson , and S. Kasen . 2008. “Comorbid Axis I and Axis II Disorders in Early Adolescence: Outcomes 20 Years Later.” Archives of General Psychiatry 65, no. 6: 641–648. 10.1001/archpsyc.65.6.641. 18519822

[pmh70041-bib-0012] d'Huart, D. , S. Seker , D. Bürgin , et al. 2023. “Key Insights From Studies on the Stability of Personality Disorders in Different Age Groups.” Frontiers in Psychiatry 14: 1109336. 10.3389/fpsyt.2023.1109336. 37398598 PMC10309036

[pmh70041-bib-0013] Dibaj, I. S. , A. J. Tørmoen , O. Klungsøyr , et al. 2024. “Early Remission of Deliberate Self‐Harm Predicts Emotion Regulation Capacity in Adulthood: 12.4 Years Follow‐Up of a Randomized Controlled Trial of Adolescents with Repeated Self‐Harm and Borderline Features.” European Child & Adolescent Psychiatry 34: 1837–1848. 10.1007/s00787-024-02602-8. 39470788 PMC12198333

[pmh70041-bib-0014] First, M. B. , J. B. W. Williams , L. Benjamin , and R. L. Spitzer . 2016. Structured Clinical Interview for DSM5 Personality Disorders (SCID‐5‐PD). American Psychiatric Publishing.

[pmh70041-bib-0015] Grilo, C. M. , C. A. Sanislow , J. G. Gunderson , et al. 2004. “Two‐Year Stability and Change of Schizotypal, Borderline, Avoidant, and Obsessive‐Compulsive Personality Disorders.” Journal of Consulting and Clinical Psychology 72, no. 5: 767–775. 10.1037/0022-006X.72.5.767. 15482035 PMC3289406

[pmh70041-bib-0016] Gunderson, J. G. 1994. “Building the Structure for the Borderline Construct.” Acta Psychiatrica 379, no. suppl: 12–18. 10.1111/j.1600-0447.1994.tb05812.x 8010146

[pmh70041-bib-0017] Gunderson, J. G. 2009. “Borderline Personality Disorder: Ontogeny a Diagnosis.” American Journal of Psychiatry 166: 530–539. 19411380 10.1176/appi.ajp.2009.08121825 PMC3145201

[pmh70041-bib-0018] Gunderson, J. G. , M. T. Daversa , and C. M. Grilo . 2006. “Predictors of 2‐Year Outcome for Patients With Borderline Personality Disorder.” American Journal of Psychiatry 163: 822–826. 16648322 10.1176/ajp.2006.163.5.822

[pmh70041-bib-0051] Gunderson, J. G. , R. L. Stout , T. H. McGlashan , et al. 2011. “Ten‐Year Course of Borderline Personality Disorder: Psychopathology and Function From the Collaborative Longitudinal Personality Disorders Study.” Archives of General Psychiatry 68, no. 8: 827–837. 21464343 10.1001/archgenpsychiatry.2011.37 PMC3158489

[pmh70041-bib-0019] Gunderson, J. G. , R. L. Stout , M. T. Shea , et al. 2014. “Interactions of Borderline Personality Disorder and Mood Disorders Over 10 Years.” Journal of Clinical Psychiatry 75: 829–834. 10.4088/JCP.13m08972. 25007118

[pmh70041-bib-0020] Hastrup, L. H. , P. Jennum , R. Ibsen , J. Kjellberg , and E. Simonsen . 2020. “Welfare Consequences of Early‐Onset Borderline Personality Disorder: A Nationwide Register‐Based Case–Control Study.” European Child & Adolescent Psychiatry 31: 253–260. 10.1007/s00787-020-01683-5. 33231787

[pmh70041-bib-0052] Hastrup, L. H. , P. Jennum , R. Ibsen , J. Kjellberg , and E. Simonsen . 2022. “Welfare Consequences of Early‐Onset Borderline Personality Disorder: A Nationwide Register‐Based Case‐Control Study.” European Child & Adolescent Psychiatry 31, no. 2: 253–260. 33231787 10.1007/s00787-020-01683-5

[pmh70041-bib-0021] Hastrup, L. H. , P. Jennum , R. Ibsen , J. Kjellberg , and E. Simonsen . 2024. “Borderline Personality Disorder and Diagnostic Co‐Occurrence of Mental Health Disorders and Somatic Diseases: A Controlled Prospective National Register‐Based Study.” Acta Psychiatrica Scandinavica 149: 124–132. 38072006 10.1111/acps.13642

[pmh70041-bib-0023] Herman, J. L. , J. C. Perry , and B. A. vanb der Kolk . 1989. “Childhood Trauma in Borderline Perdsonality Disorder.” American Journal of Psychiatry 146: 490–496. 2929750 10.1176/ajp.146.4.490

[pmh70041-bib-0024] Hutsebaut, J. , and A. Aleva . 2021. “The Identification of a Risk Profile for Young People With Borderline Personality Pathology: A Review of Recent Literature.” Current Opinion in Psychology 37: 13–20. 32653538 10.1016/j.copsyc.2020.06.004

[pmh70041-bib-0025] Johnson, J. G. , P. Cohen , J. Brown , E. M. Smailes , and D. Bernstein . 1999. “Childhood Maltreatment Increases Risk for Personality Disorders During Early Adulthood.” Archives of General Psychiatry 56: 600–606. 10401504 10.1001/archpsyc.56.7.600

[pmh70041-bib-0027] Jørgensen, M. S. , L. Møller , S. Bo , et al. 2024. “The Course of Borderline Personality Disorder From Adolescence to Early Adulthood: A 5‐Year Follow‐Up Study.” Comprehensive Psychiatry 132: 152478. 10.1016/j.comppsych.2024.152478. 38522259

[pmh70041-bib-0028] Jørgensen, M. S. , O. J. Storebø , S. Bo , et al. 2020. “Mentalization‐Based Treatment in Groups for Adolescents With Borderline Personality Disorder: 3‐ and 12‐Month Follow‐Up of a Randomized Controlled Trial.” European Child & Adolescent Psychiatry 30: 0123456789. 10.1007/s00787-020-01551-2. 32388627

[pmh70041-bib-0029] Kaess, M. , M. Thomson , S. Lerch , et al. 2024. “Age Dependent Effects of Early Intervention in Borderline Personality Disorder in Adolescents.” Psychological Medicine 54: 2033–2041. 10.1017/S0033291724000126. 38343374 PMC11413336

[pmh70041-bib-0030] Mundt, J. C. , I. M. Marks , M. K. Shear , and J. H. Greist . 2002. “The Work and Social Adjustment Scale: A Simple Measure of Impairment in Functioning.” British Journal of Psychiatry 180: 461–464. 10.1192/bjp.180.5.461 11983645

[pmh70041-bib-0031] Paris, J. , H. Zweig‐Frank , and H. Guzder . 1993. “The Role of Psychological Risk Factors in Recovery From Borderline Personality Disorder.” Comprehensive Psychiatry 34, no. 6: 410–413. 8131386 10.1016/0010-440x(93)90067-e

[pmh70041-bib-0033] Robins & Guze . 1970. “Etashilment of Diagnosic Validity.” American Journal of Psychiatry 126, no. 7: 107–111. 10.1176/ajp.126.7.983 5409569

[pmh70041-bib-0034] Shah, R. , and M. C. Zanarini . 2018. “Comorbidity of Borderline Personality Disorder: Current Status and Future Directions.” Psychiatric Clinics of North America 41, no. 4: 583–593. 10.1016/j.psc.2018.07.009. 30447726

[pmh70041-bib-0035] Sharp, C. , and A. G. Wright . 2015. “The Structure of Personality Pathology: Both a General (‘G’) and Specific (‘S) Factors.” Journal of Abnormal Psychology 124, no. 2: 387–398. 25730515 10.1037/abn0000033

[pmh70041-bib-0036] Shea, M. T. , R. L. Stout , S. Yen , et al. 2004. “Associations in the Course of Personality Disorders and Axis I Disorders Over Time.” Journal of Abnormal Psychology 113, no. 4: 499–508. 10.1037/0021-843X.113.4.499. 15535783 PMC3274820

[pmh70041-bib-0037] Sheehan, D. V. , Y. Lecrubier , K. H. Sheehan , et al. 1998. “The Mini‐International Neuropsychiatric Interview (M.I.N.I.): The Development and Validation of a Structured Diagnostic Psychiatric Interview for DSM‐IV and ICD‐10.” Journal of Clinical Psychiatry 59, no. Suppl 2: 22–33;quiz 34‐57. http://www.ncbi.nlm.nih.gov/pubmed/9881538. 9881538

[pmh70041-bib-0038] Simonsen, E. , M. Vestergaard , O. J. Storebø , S. Bo , and M. S. Jørgensen . 2021. “Prediction of Treatment Outcome of Adolescents with Borderline Personality Disorder: A 2 Year Follow‐Up Study.” Journal of Personality Disorders 35: 111–130. 33999658 10.1521/pedi_2021_35_524

[pmh70041-bib-0039] Soloff, P. H. , K. G. Lynch , and T. M. Kelly . 2002. “Childhood Abuse as a Risk Factor for Suicidal Behaviour in Borderline Patients.” JPD 16: 201–214. 10.1521/pedi.16.3.201.22542 12136678

[pmh70041-bib-0040] Stone, M. H. 1990. The Fate of Borderline Patients. Guilford Press.

[pmh70041-bib-0041] Stone, M. H. 2017. “Borderline Patients: 25 to 50 Years Later: With Commentary on Outcome Factors.” Psychodynamic Psychiatry 45, no. 2: 259–296. 28590208 10.1521/pdps.2017.45.2.259

[pmh70041-bib-0042] Storebø, O. J. , J. P. Ribeiro , M. T. Kongerslev , et al. 2021. “Individual Participant Data Systematic Reviews With Meta‐Analyses of Psychotherapies for Borderline Personality Disorder.” BMJ Open 11: e047416. 10.1136/bmjopen-2020-047416 PMC8217922 34155077

[pmh70041-bib-0043] World Health Organization . 2024. “Clinical Description and Diagnostic Requirements (CDDR) for ICD_11 Manual.” Geneva World Health Organization

[pmh70041-bib-0044] World Health Organization . Division of Mental Health . 1994. “Schedules for Clinical Assessment in Neuropsychiatry: Version 2.” World Health Organization.

[pmh70041-bib-0045] Zanarini, M. C. 2000. “Childhood Experiences Associated With Development of Borderline Personality Disorder.” Psychiatric Clinics of North America 23: 89–101. 10729933 10.1016/s0193-953x(05)70145-3

[pmh70041-bib-0046] Zanarini, M. C. 2003. The Childhood Interview for DSM‐IV Borderline Personality Disorder (CI‐BPD). McLean Hospital and Harvard Medical School.

[pmh70041-bib-0047] Zanarini, M. C. , F. R. Frankenburg , J. Hennen , D. B. Reich , and G. M. Fitzmaurice . 2012. “Fluidity of Subsyndromal Phenemonelogy of Borderline Personality Disorder Over 16 Years of Prospective Follow‐Up.” American Journal of Psychiatry 173: 688–694. 10.1176/appi.ajp.2015.15081045 PMC4930411 26869248

[pmh70041-bib-0048] Zanarini, M. C. , F. R. Frankenburg , J. Hennen , D. B. Reich , and K. R. Silk . 2005. “Psychosocial Functioning of Borderline Patients and Axis II Comparison Subjects Followed Prospectively for Six Years.” Journal of PersonalityDisorders 19, no. 1: 19–29. 10.1521/pedi.19.1.19.62178 15899718

[pmh70041-bib-0049] Zanarini, M. C. , F. R. Frankenburg , J. Hennen , D. B. Reich , and K. R. Silk . 2006. “Prediction of the 10‐Year Course of Borderline Personality Disorder.” American Journal of Psychiatry 163: 827–832. 16648323 10.1176/ajp.2006.163.5.827

[pmh70041-bib-0050] Zanarini, M. C. , F. R. Frankenburg , J. Hennen , and K. R. Silk . 2003. “The Longitudinal Course of Borderline Psychopathology: 6‐Year Prospective Follow‐Up of the Phenomenology of Borderline Personality Disorder.” American Journal of Psychiatry 160, no. 2: 274–283. 10.1176/appi.ajp.160.2.274. 12562573

